# Efficacy of* Saccharothrix algeriensis* NRRL B-24137 to suppress *Fusarium oxysporum* f.sp. *vasinfectum* induced wilt disease in cotton

**DOI:** 10.7717/peerj.14754

**Published:** 2023-02-06

**Authors:** Rizwan Asif, Muhammad Hussnain Siddique, Sumreen Hayat, Ijaz Rasul, Habibullah Nadeem, Muhammad Faisal, Muhammad Waseem, Shahbaz Ahmad Zakki, Abdelghani Zitouni, Saima Muzammil

**Affiliations:** 1 Department of Microbiology, Government College University, Faisalabad, Pakistan; 2 Department of Eastern Medicine and Surgery, Qarshi University Lahore, Lahore, Pakistan; 3 Department of Bioinformatics and Biotechnology, Government College University, Faisalabad, Pakistan; 4 Institute of Plant Breeding and Biotechnology, MNS-University of Agriculture, Multan, Pakistan; 5 Department of Environmental Science, Government College University, Faisalabad, Pakistan; 6 Department of Public Health and Nutrition, The University of Haripur, Haripur, Pakistan; 7 Laboratoire de Biologie des Systèmes Microbiens (LBSM), Ecole Normale Supérieure de Kouba, Alger, Algeria

**Keywords:** *F. oxysporum* f.sp. vasinfectum, Cotton wilt, *Sa. algeriensis* NRRL B-24137, Biocontrol agent

## Abstract

*Fusarium* cotton wilt is a devastating disease of the cotton crop throughout the world, caused by *Fusarium oxysporum* f.sp. *vasinfectum* (FOV). Chemical control has many side effects, so, biological controls have been widely used for the management of *Fusarium* wilt. This study aimed to investigate the possible use of an actinomycetes *Saccharothrix algeriensis* (SA) NRRL B-24137 to control FOV. To access *in-vitro* anti-*Fusarium* ability of SA NRRL B-24137, dual culture assay, spore germination and seed germination tests were carried out. Following *in-vitro* investigations, several pot tests in a greenhouse environment were used to evaluate the biological control potential of SA NRRL B-24137 against FOV. Dual culture assay and spore germination revealed that SA NRRL B-24137 showed significant anti-*Fusarium* activity.During spore germination 87.77% inhibition of spore germination were observed. In pot experiments, SA NRRL B-24137 primed cotton seeds resulted in a 74.0% reduction in disease incidence. In soil there was a significant reduction in FOV spores in the presence of SA NRRL B-24137. Positive correlation was also observed on different concentrations of SA NRRL B-24137 towards FOV reduction. The results of this study showed that SA NRRL B-24137 has the potential to be employed as a biocontrol agent against *Fusarium* cotton wilt, improving cotton growth characteristics and yield.

## Introduction

*Fusarium oxysporum* is one of the most common soil-borne pathogens that cause wilt disease. *F. oxysporum* ranked fifth among the top ten plant pathogenic fungi, in term of economic loss and a serious threat for sustainability of agriculture (Dean et al., 2012). *F. oxysporum* causes severe damage and yield losses in many economically important agricultural crops such as cotton, tomato and chickpea (El-Hassan & Gowen, 2006; Gopalakrishnan et al., 2011; Van Der Does et al., 2008). There are different formae speciales (f.sp.) of *F. oxysporum* affecting specific hosts such as *F. oxysporum* f.sp. *lini, F. oxysporum* f.sp. *lycopersici* and *F. oxysporum* f.sp. *vasinfectum* are pathogenic to flax, tomato and cotton respectively. FOV is an important cotton crop pathogen that affects nearly all cotton growing regions of the world. Other species of *Fusarium* also have been concomitant with cotton roots but their influence in cotton production as root pathogen is still blurred (Zhang et al., 1996). According to reports, FOV is a disease of Pakistan’s largest kharif cotton crop, produced on 12% of the country’s total cultivated land and resulting in significant economic loss (Rajput et al., 2006).

An ascomycetes pathogen called *F. oxysporum* may survive in the soil for a long time in a latent state and produce chlamydospores (Bennett, 2012). In most cases, *F. oxysporum* may enter plants through their roots (Asif et al., 2020; Hillocks et al., 2000). The wilt disease causes the roots to dry out, the foliage to wilt, the leaves to wither, the shape of the plants to become stunted, and the plant’s crown to become discolored. Cortical tissues and internal vascular of plant crowns showed orange to brown discoloration (Fang, You & Barbetti, 2012; Halpern et al., 2018). Once, *F. oxysporum* enters the xylem of the roots, fungicides cannot affect the pathogen (Lamia et al., 2017). The most effective control of cotton *Fusarium* wilt has been achieved by integrated management practice like crop rotation, growing resistant varieties and soil fumigation with chemicals (Jiménez-Díaz et al., 2011). However, the high cost of developing resistant varieties and environmental concerns of chemicals make it an urgent to explore other control methods (El-Sheekh, Mousa & Farghl, 2020). Biological control agents have developed as a viable alternative method to control soil-borne illnesses in recent years (Xie et al., 2017). For sustainable agriculture, biological control appears to have supremacy. Numerous studies have demonstrated the use of antagonistic bacteria like *Streptomyces* spp., *Pseudomonas putida, Bacillus* spp. and different endophytic bacteria to protect plants from *F. oxysporum* (Slama et al., 2019; Pereg & McMillan, 2015). These bacteria reduce the disease incidence by competing to soil pathogens through antibiotic production and induction of plant resistance (Köhl, Kolnaar & Ravensberg, 2019; Compant et al., 2013a). So, biological control agents are also a better substitute to resolve this problem.

Among these microorganisms, actinobacteria (a major group of soil bacteria) revealed their biocontrol ability against soil-borne fungi, including *F. oxysporum* (Gopalakrishnan et al., 2011). Actinobacteria normally form symbiotic relationships with agricultural plants, invading their interior tissues without exhibiting disease signs and producing a significant amount of antifungal secondary biometabolites that benefit their host plant by enhancing growth and productivity (Boukaya et al., 2018; Igarashi, 2004; Khamna, Yokota & Lumyong, 2009a). *Streptomyces* (largest group of actinobacteria) widely distributed in soil have been predominantly studied due to their antibiotic-producing capacity and documented as biocontrol agents. Commercially, *Streptomyces* based fungicides have been marketed against soil-borne pathogens like *Fusarium* (Lahdenperä, Simon & Uoti, 1991; Yuan & Crawford, 1995). As *Streptomyces* capable to grow an array of secondary metabolites, they received attention for biocontrol agent against soil borne fungus in agriculture. Recently, Bubici (2018) proved the application of *Streptomyces* against *Fusarium* wilt disease.

However, among the rare actinobacteria groups, Saccharothrix has not been explored largely for their biocontrol activity. SA NRRL B-24137 is a gram positive bacterium isolated from Sahara soil, which produces numerous bioactive metabolites, belonging to pyrrothine, known as dithiolopyrrolone derivatives (Lamari et al., 2002; Merrouche et al., 2010; Merrouche et al., 2011). SA NRRL B-24137 has been reported having some antibacterial and antifungal activities (Merrouche et al., 2011; Muzammil et al., 2012; Saker, Lebrihi & Mathieu, 2014). As a whole, the prime objective of this study is the evaluation of biocontrol potential of well documented SA NRRL B-24137 for the first time against *Fusarium* wilt in cotton plants in greenhouse conditions. The results of this study may be used to create biofertilizer that will aid in environmentally friendly farming.

## Materials and Methods

### Microorganisms and culture conditions

The strain SA NRRL B-24137 used in this study was procured from Laboratoire de Biologie des Systèmes Microbiens, Ecole Normale Superieure, Kouba Algiers and maintained on slants of International Streptomyces Project 2 medium (ISP-2) at 4 °C (Merrouche et al., 2017).

FOV was procured from the Department of Plant Pathology, University of Agriculture, Faisalabad. FOV was grown on Potato Dextrose Agar (PDA) medium at 25 °C for 7 days (Li et al., 2018).

### *In-vitro* anti-*Fusarium* activity of SA NRRL B-24137

#### Anti-*Fusarium* activity of SA NRRL B-24137 by dual culture plate method

*In-vitro*, anti-*Fusarium* activity of SA NRRL B-24137 was evaluated using a dual-culture method containing half of the PDA and half of the ISP2 media. FOV with 10^6^ spore/ml concentration were streaked towards the margin of the Petri dish while the culture of SA NRRL B-24137 was streaked in the center of Petri dish and incubated at 25 °C ± 2 °C for 7 days. The zone of inhibition between the colony margin of SA and FOV was calculated to ascertain the antifungal activity of SA NRRL B-24137 (Hilaire et al., 2015).

#### Effect of SA NRRL B-24137 on spore germination of FOV

SA NRRL B-24137 antifungal effect on the spore germination of FOV was investigated using the method of Ahmed (2010). FOV spore suspension with concentration of 10^6^/ml (500 µL) was added to SA NRRL B-24137 suspension (500 µL) in glass vials (30 ml) and incubated at 25 °C for 24 h. After 24 hrs incubation, spores in the vials were stained with lactophenol cotton blue and observed for germination status under a microscope. Percentage inhibition was calculated as % Spore inhibition =*A* − *B*/*A* × 100 whereas A: Spore germination in control; and B: Spore germination in treatment.

#### Anti-*Fusarium* effect of SA NRRL B-24137 on seed germination

Disease-free, fresh cotton seeds were taken from Ayub Agricultural Research Institute, Faisalabad, Pakistan. Seeds were soaked for 3 min in H_2_SO_4_ for surface sterilization and rinsed with distilled water in triplicate. After sterilization cotton seeds (*n* = 07) were placed in a Petri dish containing FOV culture (10^6^ spore/ml) and an equal number of seeds were also placed in a Petri dish containing a mixture of both SA NRRL B-24137 (10^7^ cfu/ml) and FOV (10^6^ spore/ml) culture each with same concentration (500 µL). Both plates were cultured at 25 °C ± 2 °C for 24 h. Then seeds were separated from the culture plates and, after washing, transferred to the sterilized Petri plates containing a double layer of wet blotter paper. After 7 days of incubation, average seed germination was observed in both plates following the method of Jain & Choudhary (2014).

### *In-vivo* anti-*Fusarium* activity of SA NRRL B-24137

#### Seed treatment

Sterilized and disease-free, fresh cotton seeds were soaked for 18 h in bacterial SA suspension (10^7^ cfu/ml). In control , seeds were soaked only in carboxymethyl cellulose (CMC) solution. In a sterile setting, a laminar flow hood was used to dry the seeds. Dilution plating suspensions of 10 seeds in 10 ml PB on ISP2 medium yielded a mean bacterial concentration of 1 × 10^7^, which was used to calculate the amount of CFU of bacteria per seed (Erdogan & Benlioglu, 2010).

#### Biological control of *Fusarium* wilt in the greenhouse

Seeds treated with bacterial suspension (10^7^ cfu/ml) and control seeds were sown in pots containing autoclave soil (100 g/pot). With the use of a 22-gauge needle, 10 µL conidial suspension of FOV (10^6^ spore/ml) prepared using Hanson’s (2000) technique was injected into the plant body above the soil at the level of the first internode at the six true leaves stage. One drop was also injected into the stele of cotton. At the same time, sterile water is used for the control plants. Pots placed at 27 °C ± 2 °C in the growth room. A scale containing 5 grade (0-4) was used to measure the disease severity both in bacterial treated and control plants (Song et al., 2004).

Disease severity (DS) was determined using equation,

\begin{eqnarray*}\text{Disease Severity}= \frac{ \left( \Sigma ~\text{rating no}. \right) \left( \mathrm{no}. \text{plants in rating category} \right) }{ \left( \text{Total no}. \text{plants}  \right) \left( \text{highest rating value} \right) } \times 10 \end{eqnarray*}

#### Evaluation of SA NRRL B-24137 biocontrol ability

For six weeks, pot tests were conducted to assess SA NRRL B-24137’s antagonistic effect on FOV. Sterilized seeds were planted in pots (10 seeds/pot, one cm depth) filled with soil that had already been pre-inoculated with FOV (10^6^ conidia/gds), which served as the control group, for this activity. In the experimental group, seeds were sown in pots holding soil pre-inoculated with SA NRRL B-24137 suspension (10^7^ CFU/gds) and FOV (10^6^ conidia/gds). The pots were placed for 3 days in a dark place for maximum germination of seed in the growth chamber.

#### Evaluation the effect of FOV and SA NRRL B-24137 at different concentrations

The disease severity at various concentrations of FOV (0, 10, 10^2^, 10^3^, 10^4^, 10^5^–10^6^ conidia/mL) in cotton plants was also scrutinized. Sterilized and healthy seeds were implanted in the earthen pot. When seedlings reached the three true leaves stage, plants were treated with a 25 mL spore suspension of FOV with different spore concentrations (0, 10, 10^2^, 10^3^, 10^4^, 10^5^–10^6^). All plants were placed in the greenhouse at optimum conditions for 45 days to observe the disease severity and incidence. The experiments were arranged in the following order,

Group 1: Plants without pathogen FOV (control)

Group 2: Plants were infected with 10 spore/ml dose of pathogen FOV

Group 3: Plants inoculated with 10^2^ spore/ml dose of FOV

Group 4: Plants were provided with 10^3^ spore/ml dose of FOV

Group 5: Plants were given with 10^4^ spore density/ml dose of FOV

Groups 6: Plants were provided with a pathogen dose 10^5^ spore/ml,

Group 7: Plants were provided 10^6^ spore/ml dose of pathogen

To assess anti-*Fusarium* potential of SA NRRL B-24137, different concentrations of SA NRRL B-24137 suspension (0-10^7^ CFU/mL) were prepared, and seeds were primed with different concentrations for 18 h before sowing in the pot. Plants were infected with various concentrations (10^4^–10^6^) of FOV when these plants reached at three true leaves stage. Disease severity was observed after 45 days of treatment.

#### Determination of SA NRRL B-24137 and FOV population density in the soil

The population density of SA NRRL B-24137 and FOV in soil was determined up to 9 weeks. SA NRRL B-24137 (10^7^ CFU/gds) was added to the soil, and after 24 h, FOV (10^6^ spore/gds) was also introduced. The serial dilutions of soils on nutrient agar were used to establish the microbial population density in the soil. The colonies of FOV and SA NRRL B-24137 were observed and counted after 3 and 7 days of incubation at 25 °C. Different antibiotics such as 25 mg/L kanamycin, 10 mg/L streptomycin and 50 mg/L chloramphenicol were added to the medium to prevent unwanted microbial growth.

#### Effect of SA NRRL B-24137 on plant growth

SA NRRL B-24137 effect on different growth parameters of cotton plants was evaluated against FOV. After 45 days of post inoculation, plants were uprooted from the pots and recorded the vegetative growth parameters (plant height (cm), plant weight (gm), root length (cm)). Four treatments were made with three plants in each treatment. Following treatments were used for this experiment.

Treatment 1: Uninoculated control

Treatment 2: FOV

Treatment 3: SA NRRL B-24137 + FOV

Treatment 4: SA NRRL B-24137

After performing all experiments in greenhouse, infected plants and soil were autoclaved before waste to avoid the spread of infection.

#### Data analysis

Every experiment was carried out in triplicate. The mean ± standard error of the mean is used to express data. GraphPad Prism 5.0 was used to conduct the statistical analysis (Graphpad Prism Software, San Diego, CA, USA). After the analysis of variance, Tukey’s test is conducted. Statistics were judged to be significant for all *p*-values under 0.05.

## Results

### *In-vivo* anti-*Fusarium* activity of SA NRRL B-24137

#### Anti-*Fusarium* activity of SA NRRL B-24137 by dual culture plate method

**Figure 1:**
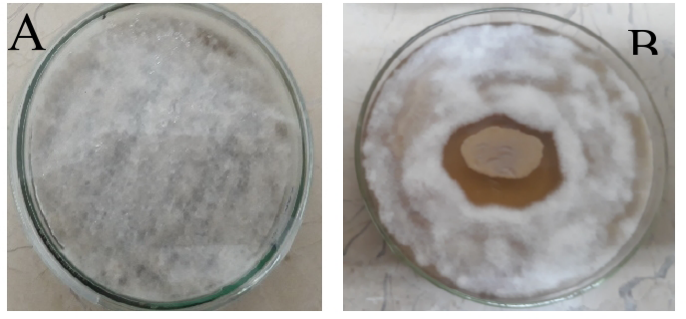
*In-vitro* antifungal activity of *Sa. algeriensis* against *F. oxysporum* on ISP-2 medium for 7 days at 27 + 2 °C. (A) *F. oxysporum* alone. (B) *Sa. algeriensis* with *F. oxysporum* showing the zone of inhibition.

*In-vitro* dual culture plate method was used to determine the anti-*Fusarium* activity of SA NRRL B-24137. The results revealed that SA NRRL B-24137 showed significant antifungal activity against FOV as depicted in Fig. 1. The growth of FOV on a dual culture plate and on control plate (FOV alone) was compared against zone of inhibition.

#### Effect of SA NRRL B-24137 on spore germination of *F. oxysporum*

**Figure 2:**
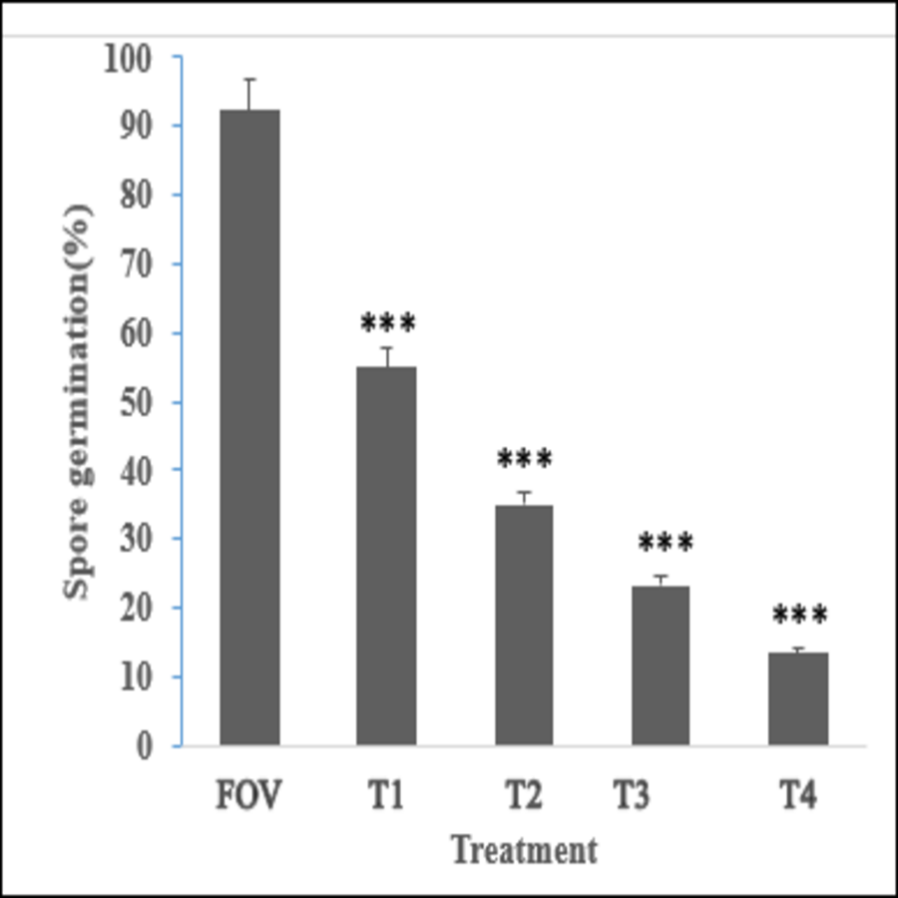
Inhibitory effect of SA NRRL B-24137 on spore germination of FOV (10^6^ spore/mL). Positive correlation was observed with increased dose of SA NRRL B-24137. Where *T*_1_ = (SA 10^7^ CFU/mL), *T*_2_ = (SA 10^6^ CFU/mL), *T*_3_ = (SA 10^5^ CFU/mL) and *T*_4_ = (SA 10^4^ CFU/mL). ***p < 0.001 compared to FOV group.

SA NRRL B-24137 antifungal effect on the FOV spore germination was examined. The obtain results showed that SA NRRL B-24137 reduced the spore germination. A positive correlation was also determined with increase the Colony Forming Unit (CFU) of SA NRRL B-24137 the inhibition of FOV spore germination was also increased. SA NRRL B-24137 at 10^7^ CFU/mL gave maximum percentage of FOV spore germination inhibition (87.77%) followed by 10^6^, 10^5^ and 10^4^ CFU/mL as shown in (Fig. 2).

#### Anti-*Fusarium* effect of SA NRRL B-24137 on seed germination

**Figure 3:**
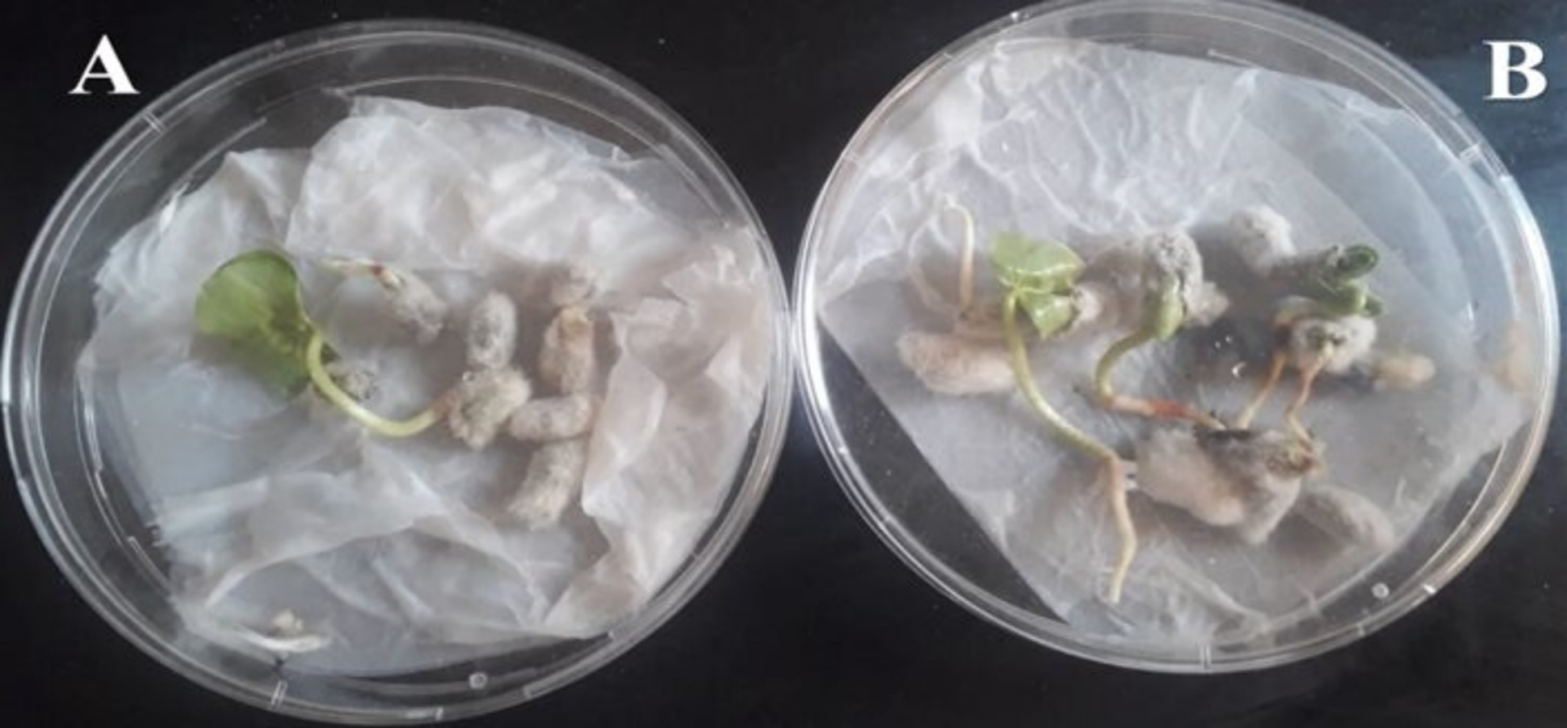
Effect of SA on seed germination against FOV. (A) Petri plate containing seeds treated with FOV showed minimum seed germination. (B) Petri plate containing seeds treated with SA & FOV with maximum seed germination. Both control and experimental Petri plates were placed at 27 °C ± 2 °C for 7 days and results were observed.

Effect of SA NRRL B-24137 on seed germination against FOV was evaluated and results exhibited that FOV have high degree of pathogenicity. An average 25% seed germination was observed in Petri plates containing FOV treated seeds relative to Petri plates containing SA NRRL B-24137 + FOV treated seed with an average of 80% seed germination (Fig. 3).

### *In-vivo* anti-*Fusarium* activity of SA NRRL B-24137 (SA)

#### Exploitation of antifungal potential of SA NRRL B-24137 under greenhouse conditions

**Figure 4:**
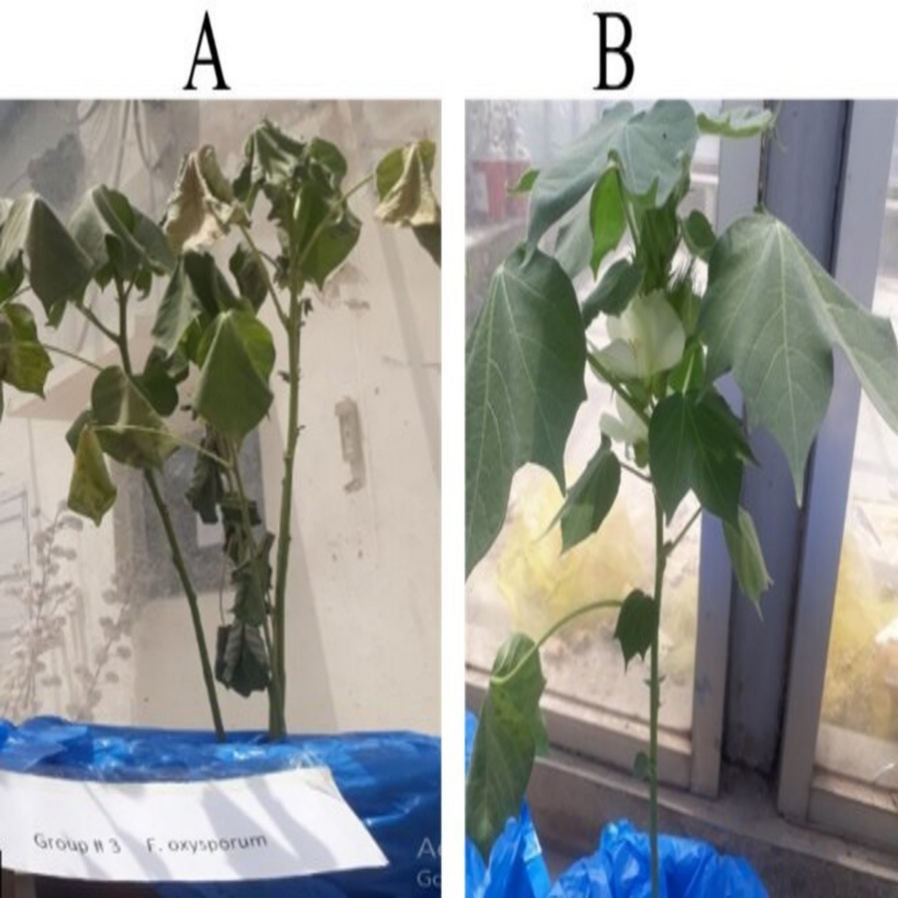
Effect of seed priming with SA NRRL B-24137 towards *Fusarium* cotton wilt. (A) Plants infected with FOV showed the symptoms of wilt. (B) Plants treated with SA NRRL B-24137 and infected with FOV were healthy in appearance. Both control and experimental pots were placed in greenhouse with a favorable environment for 21 days.

After evaluating *in-vitro* anti-*Fusarium* potential, the *in-vivo* efficiency of the SA NRRL B-24137 strain against *Fusarium* wilt in cotton plants was evaluated under greenhouse conditions. Suspension of SA NRRL B-24137 was prepared and diluted according to requirement, before sowing, cotton seeds were dipped in SA NRRL B-24137 suspension for 18 h. The plants infected with the FOV pathogen showed wilt symptoms after 21 days (Fig. 4A). At the same time, the plants treated with SA NRRL B-24137 before being challenge with the FOV pathogen were healthy in appearance (Fig. 4B).

**Figure 5:**
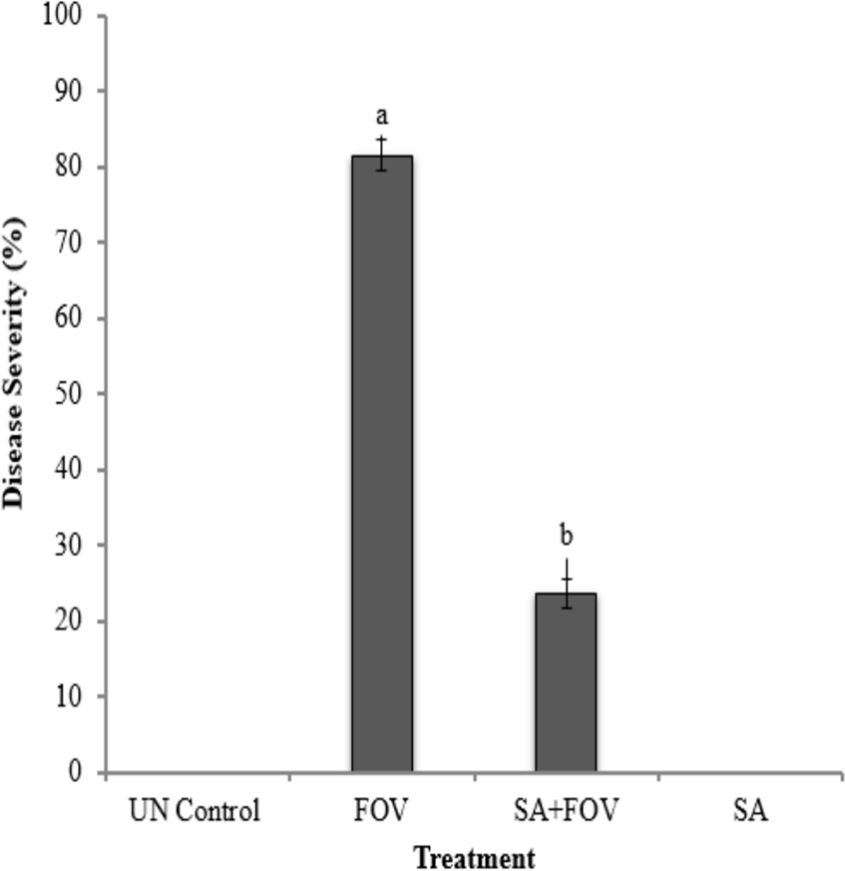
Antagonistic effect of pre-sowing seed treatment with the suspension of SA on *Fusarium* cotton wilt disease severity. FOV represent *F. oxysporum* f.sp. *vasinfectum* and SA represent *Sa. algeriensis*. The bar indicates the standard deviation of the mean (*n* = 3). ^a^ *p* < 0.001 compared to the UN control group. ^b^ *p* < 0.001 compared to the FOV group.

Disease incidence in the SA NRRL B-24137 treated and challenged with FOV plants was approximately 23%, which was significantly lower than the FOV infected control plants (81%) with rapid wilting of cotton leaves. Specifically, treatment of cotton plants with SA NRRL B-24137 resulted in >74.0% suppression of *Fusarium* wilt. ANOVA was performed to analyze the results, and a significant difference was observed between the experimental and control groups (Fig. 5).

#### Evaluation of SA NRRL B-24137 biocontrol ability

**Figure 6:**
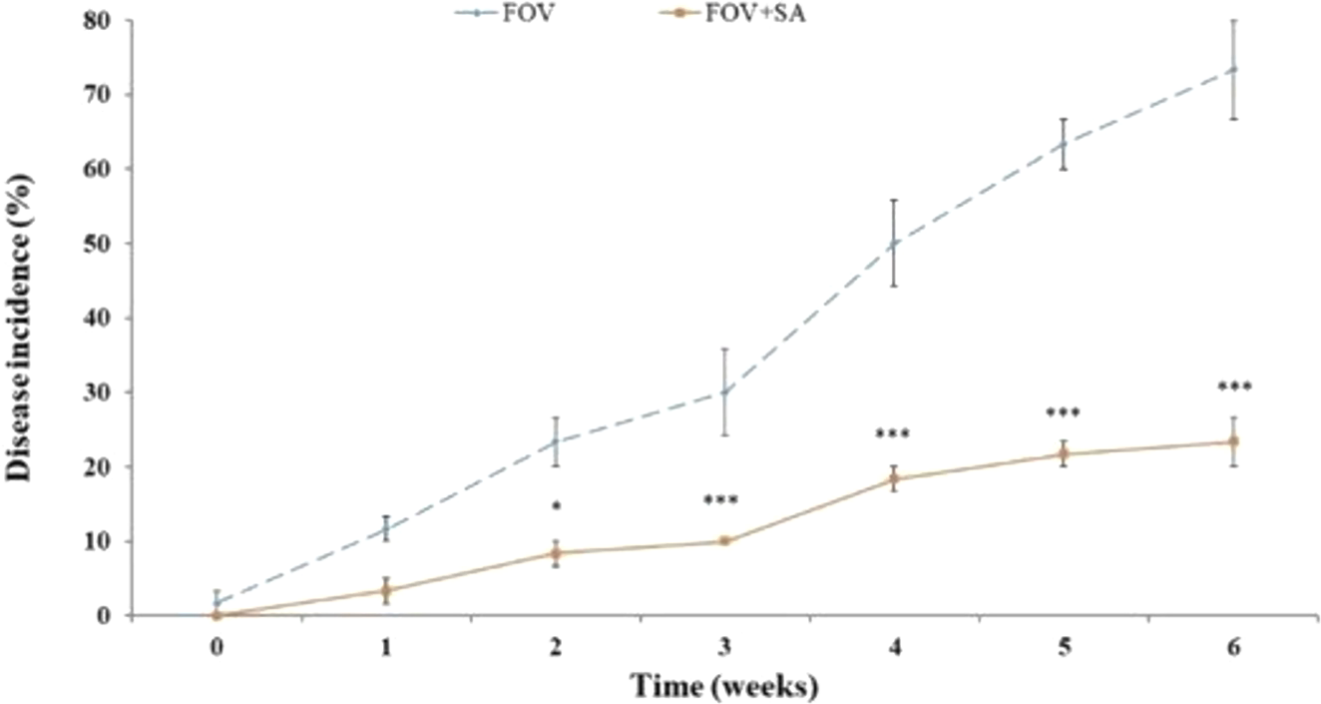
*Sa. algeriensis* NRRL B-24137 (SA) can control *F. oxysporum* f.sp. *vasinfectum* (FOV) induced wilt in cotton plants. Greyline indicates *Fusarium* wilt of cotton in soil infested with FOV (control group): dark line indicates plants grown in soil treated with SA and FOV (experiment group). SA inoculated plants exposed that disease incidence reduced progressively week by week (1–6 week) as compared to FOV infected cotton plants. Bars indicate standard deviation of the mean (*n* = 3). *p < 0.05 & ***p < 0.001 compared to the FOV group.

The biocontrol potential of SA NRRL B-24137 on disease severity against FOV was recorded up to 6 weeks, and obtained result reveal the remarkable antifungal potential of SA NRRL B-24137 with decreased disease severity gradually in SA NRRL B-24137 treated plants compared to control group (FOV) as shown in (Fig. 6). SA NRRL B-24137 remarkably lowered the soil colonization of pathogen FOV in the pot experiment and subsequently decreased disease incidence over time compared to FOV infected control plants.

**Figure 7:**
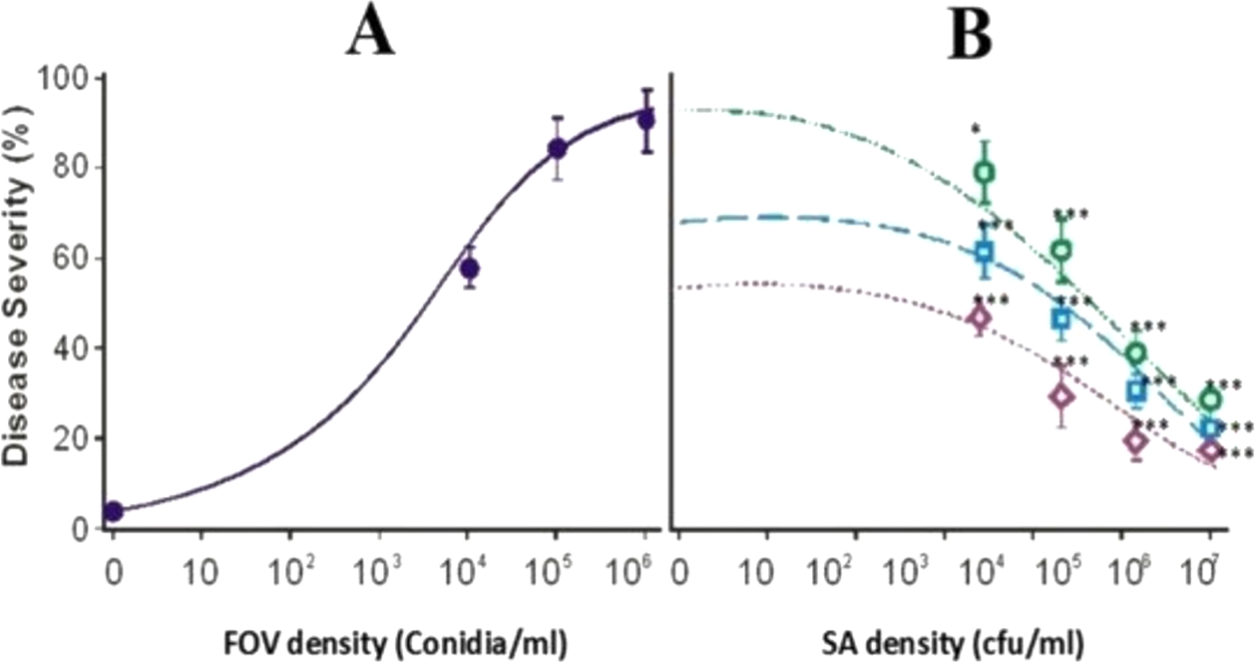
The disease severity of *Fusarium* wilt of cotton was observed with and without antagonists under greenhouse conditions. (A) Disease severity of cotton wilt was increased with increased pathogen *F*. *oxysporum* f.sp. *vasinfectum* dose (without antagonistic) (B) Decline trend of disease severity in cotton wilt was observed with increased antagonistic *Sa. algeriensis* inoculum dose against different pathogen doses; the green line showed *Fusarium* 10^6^ conidial spore/mL, the blue line showed *Fusarium* 10^5^ conidial spore/ml and the purple line showed *Fusarium* 10^4^ conidial spore/ml. FOV, *F*. *oxysporum* f.sp. *vasinfectum* and SA, *Sa. Algeriensis.* *p < 0.05 & ***p < 0.001 compared to A group.

After evaluating the effect of FOV and SA NRRL B-24137 on their different concentrations, it was shown that as pathogen FOV dosage (0–10^6^ spore/mL) grew, so did the severity of the disease (Fig. 7A). Moreover, the antagonistic effect of SA NRRL B-24137 was also observed with various concentrations (0–10^7^ CFU/mL) against FOV at 10^4^–10^7^ spore/mL A positive correlation was also found (Fig. 7B). It was confirmed that SA NRRL B-24137 is an effective agent for suppressing *Fusarium* wilt of cotton caused by FOV but its efficiency differed depending on the concentrations of SA NRRL B-24137 suspension.

#### Determination of SA NRRL B-24137 and FOV population density in the soil

**Figure 8:**
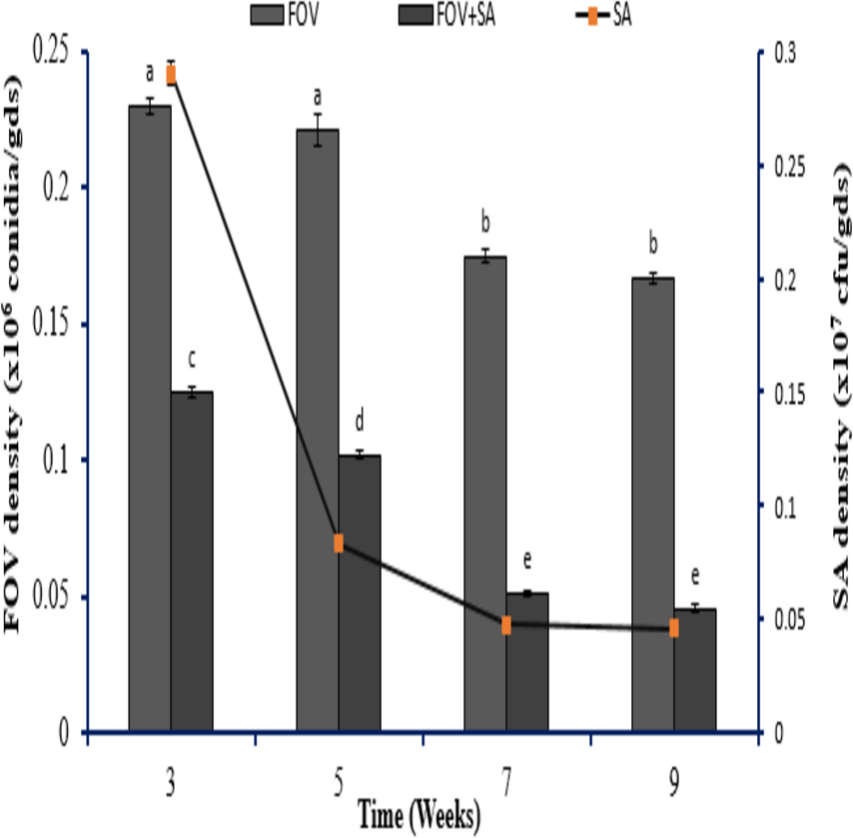
Assessment of *Sa. algeriensis* NRRL B-24137 and *F. oxysporum* f.sp. *vasinfectum* population density in the soil. Different population densities of *Sa. algeriensis* NRRL B-24137 (10^8^ cfu/gds) and *F. oxysporum* f.sp.

**Figure 9:**
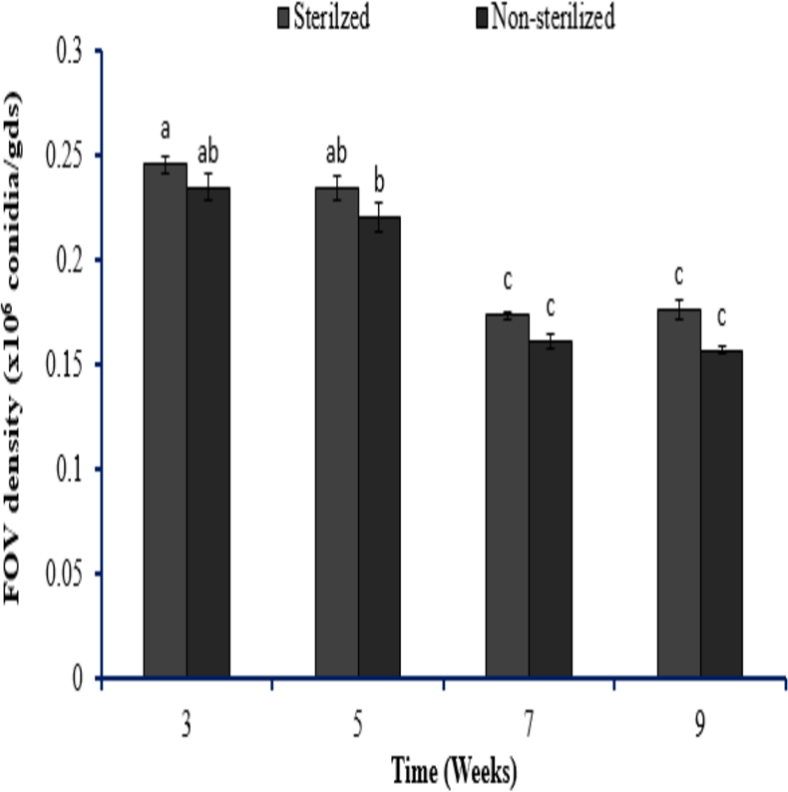
Evaluation of *F. oxysporum* f.sp. *vasinfectum* papulation density in the sterilized and non-sterilized soil from 3 to 9-week experiment. Initial dose of *F. oxysporum* f.sp. *vasinfectum* 10^6^ conidia/gds was introduced into the soil. The bars indicates the standard deviation of the mean (*n* = 3). Different letters represent significant differences among treatments (*p* > 0.05).

To assess SA NRRL B-24137 anti-*Fusarium* activity toward FOV in soil, pot tests were conducted. The initial density of SA NRRL B-24137 (10^7^ CFU/gds) and FOV (10^6^/gds) was introduced into the natural soil. Results showed that SA NRRL B-24137 treatment significantly decreased the FOV soil density and SA NRRL B-24137 remained stable after 9 weeks of 9 weeks” experiment (Fig. 8). In both the sterilized and unsterilized soil, FOV density was calculated, but no discernible change was found (Fig. 9). These findings proved that SA NRRL B-24137 was only responsible for reducing FOV density in the soil and the soil microflora did not interfere in the FOV soil population.

#### Plant growth promotion (PGP) parameters

**Figure 10:**
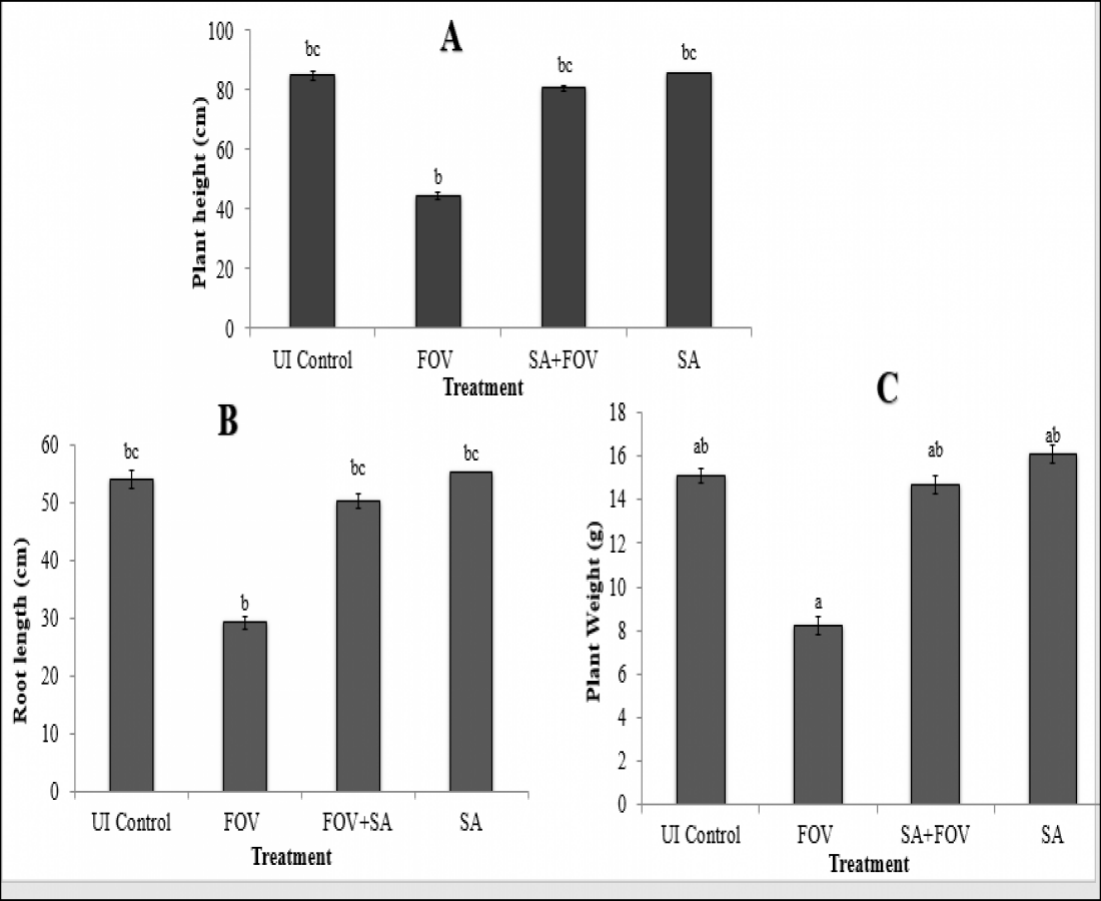
Effect of seed treatment with *Sa. algeriensis* on vegetative plant growth parameters such as Plant height, root length and plant weight of cotton after 45 days in a greenhouse. Plants treated with *Sa. algeriensis* show more growth parameters relative to *F. oxysporum* infected plants. Different letters in the respective bar are significantly different, and columns with the same letter are not significantly different. Values shown are the means ± SEs errors obtained from three replicates.

**Figure 11:**
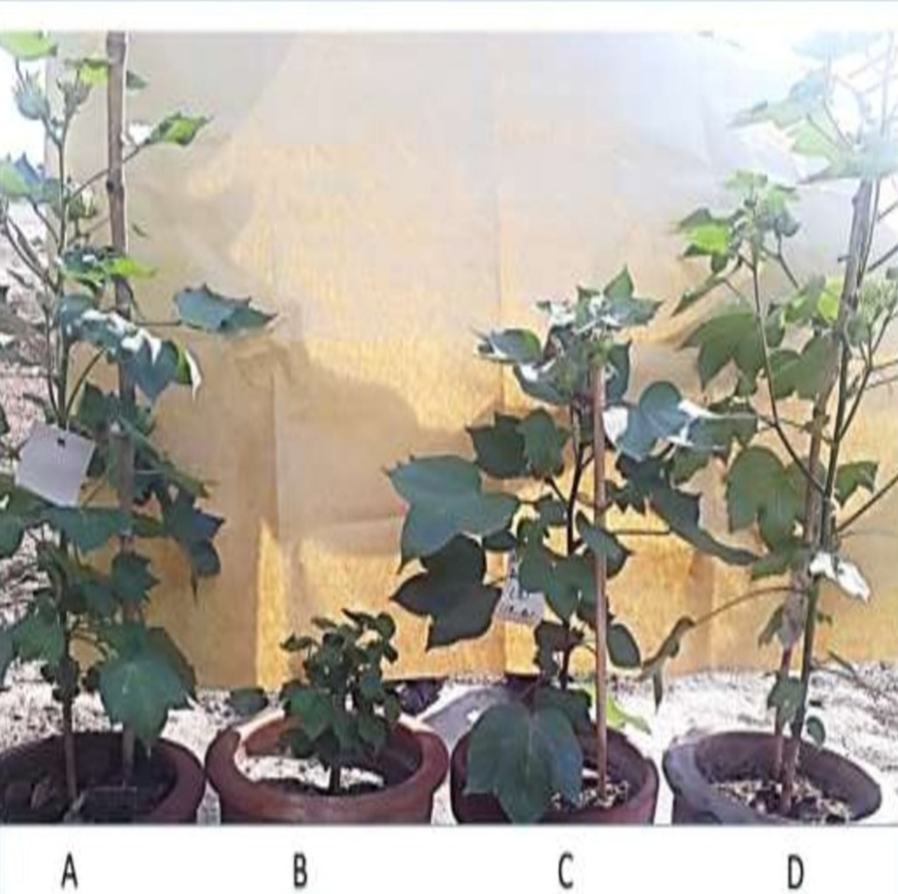
Effect of SA NRRL B-24137 seed treatment on *Fusarium* wilt incidence and growth of cotton plants in pot experiment. (A) Uninoculated control, (B) FOV pathogen-infected plants, (C) SA NRRL B-24137 treated plants and challenged with FOV and D: SA NRRL B-24137 treated plant.

**Figure 12:**
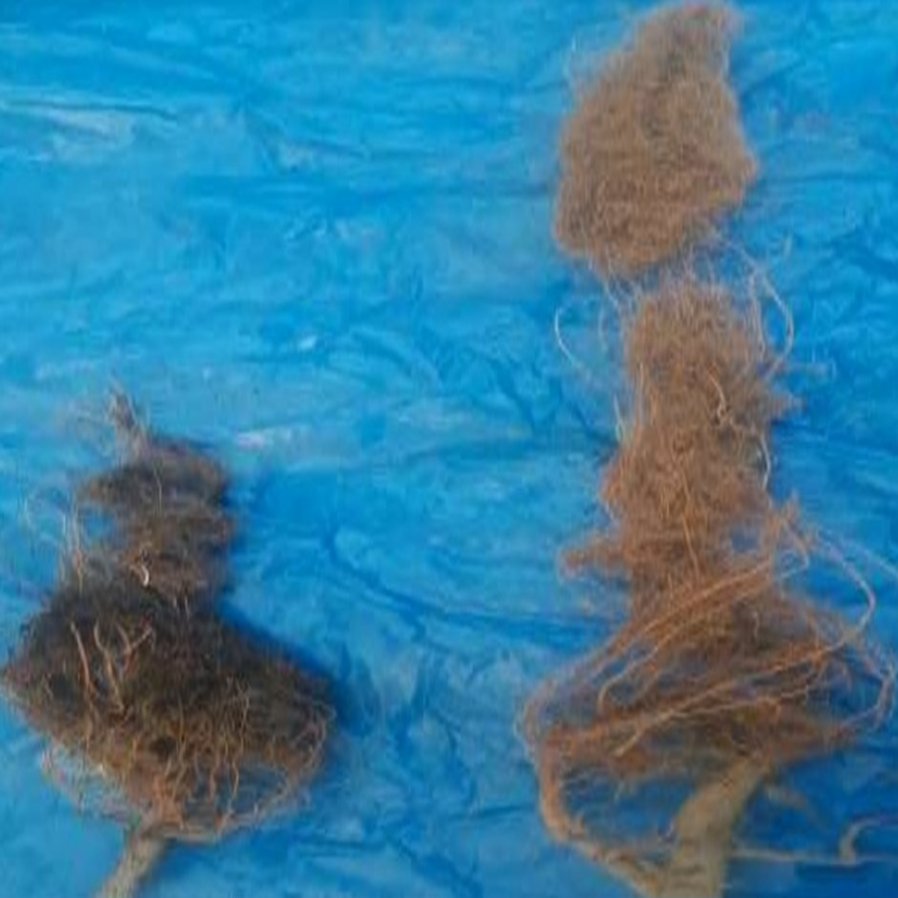
Effect of *Fusarium* on roots of cotton plants compared to healthy plants after 45 days of post inoculation of FOV. (A) Roots of cotton plants infected with FOV pathogen showed blackish brown lesions with reduced length; (B) plants treated with SA healthy roots showed no symptoms and increased root length.

During greenhouse experiments, different growth parameters like plant weight, plant length and roots length were also recorded. After 45 days of plantation, plant height, root length and fresh weight of SA NRRL B-24137 treated plants were average 80.67 cm, 50.27 cm, and 14.69 g, respectively while in control plants 44.47 cm plant height, 29.20 cm root length and 8.21 g plant weight, respectively (Fig. 10). Surprisingly, all growth parameters were found higher in only SA NRRL B-24137 treated plants compared to all other groups (Figs. 10 and 11). This might be due to its PGPR activities. Meanwhile, root infected with FOV also differs in morphology as compared to roots morphology of SA NRRL B-24137 treated plants (Fig. 12).

## Discussion

One of the most significant commercial crops in the world, is cotton (*Gossypium hirsutum* L.). *Fusarium* wilt, caused by FOV(a pathogen that causes severe losses in cotton). Recent study was performed to assess the biocontrol potential of an actinobacterium, *i.e.,* SA NRRL B-24137 both *in-vitro* and *in-vivo* conditions against FOV.

*In-vitro* evaluation of SA NRRL B-24137 confirmed anti-*Fusarium* activity and in literature, *Bacillus* spp. have been widely used as biocontrol agents to suppress *Fusarium* wilt disease (Bhattacharyya & Jha, 2012; Gowtham et al., 2016). Cavaglieri et al. (2005) also used *B. subtilis* in maize plants against *Fusarium verticillioides* and found a significant decrease in the disease. Previous studies demonstrated that chitinolytic bacteria are also be able to prevent *Fusarium* wilt in tomatoes and cucumber (Hariprasad, Divakara & Niranjana, 2011; Singh et al., 1999). Numerous studies have proved the efficacy of endophytic bacteria as a biocontrol agents for cotton wilt (Chen et al., 1995; Compant et al., 2013b).

Antifungal activity of *Saccharothrix species* against a variety of fungi, including *Aspergillus, Fusarium, Penicillium*, and *Ascochyta* have been reported (Boubetra et al., 2013; Zitouni et al., 2005). *In-vitro* biocontrol ability of SA NRRLB-24137 against *Botrytis cinerea* and also in grapevine plants has been reported (Compant et al., 2013b; Muzammil et al., 2012). One of the reason for SA NRRL B-24137 having significant antifungal properties is its ability to colonize the roots of plants (Compant et al., 2013b). Additionally, SA NRRL B-24137 is known to produce dithiolopyrrolones, which are bioactive substances thought to have antifungal activities (Merrouche et al., 2010; Merrouche et al., 2011). Dithiolopyrrolones produced by actinobacteria other than SA NRRL B-24137, such as *Streptomyces*, also exhibited antifungal activities towards filamentous fungi including *Botrytis*, *Fusarium*, *Aspergillus*, *Rhizoctonia*, *Phytophthora*, *Verticillium*, *Penicillium* and *Trichoderma* (Chen, Dunphy & Webster, 1994; Khamna et al., 2009b).

The role of *Fusarium* as biocontrol agents in the proximity to the roots is suggested as the most effective strategy by the researcher (Deketelaere et al., 2017). In particular, seed treatment could be an efficient technique to provide better relief from subsequent *Fusarium* infection (Chen et al., 1995). In this study, above 74% disease incidence inhibition and a considerable decline in disease severity was observed in SA NRRL B-24137 treated plants. These experiments demonstrated the effectiveness of SA NRRL B-24137 as a biocontrol agent against the wilt disease.

Merrouche et al. (2017) studied the soil population density of SA NRRL B-24137 and FOV up to 9 weeks, and SA NRRL B-24137 reduced the *Fusarium* soil density. SA NRRL B-24137 colonization process is also explained by Compant et al. (2013b) in grapevine plants. An essential component of antagonistic bacteria as a biocontrol agent is thought to be capacity to maintain a certain level of population density in the rhizosphere. Some researchers suggest that competition for carbon and iron is a limiting factor for pathogenic fungi growth and the production of antimicrobial compounds by biocontrol agents are involved for the antifungal activities (Getha & Vikineswary, 2002; Marzano, Gallo & Altomare, 2013). SA NRRL B-24137 is well defined actinobacteria keeping plant growth and antifungal properties (Muzammil et al., 2012). Production of secondary metabolites against pathogen is responsible for the biocontrol properties of many bacteria as in SA NRRL B-24137 documented in literature (Benítez et al., 2004; Kanini et al., 2013). Results demonstrated that cotton seed treatment with SA NRRL B-24137 significantly reduce symptoms of *Fusarium* cotton wilt.

SA NRRL B-24137 could control the pathogen by different methods like niche competition, pathogen interference, signaling, and augmenting plant defense using induced systemic resistance (Compant et al., 2013a; Muzammil et al., 2014). Previously, actinobacteria (*Streptomyces spp.*) have been demonstrated to control soil borne pathogen (*F. oxysporum*) but according to our knowledge, only study has been conducted with non-*Streptomyces* actinobacteria (SA) against *F. oxysporum* in plants (Toumatia et al., 2015; Yekkour et al., 2012). This research is the first study to demonstrate the biocontrol potential of SA NRRL B-24137 against FOV and first time SA is used for plant biocontrol in Pakistan. However, further investigations are required to evaluate SA NRRL B-24137 potential against the vascular wilt disease at field level and establish SA with other microorganisms as biocontrol agent in crops.

Biological control is a promising technology for environmentally friendly agriculture since provides an alternate method of preventing plant diseases (Kaur, Kaur & Singh, 2011). Currently, quite biocontrol agents (BCA) are discovered to efficiently manage plant diseases and are all environmentally sound evidence (Howell, 2003; Kakvan et al., 2013; Kaur, Kaur & Singh, 2011; Sang & Kim, 2014). However, the overall efficacy of any biocontrol agent depends on many factors such as environmental conditions and application methods. To exploit the potential of SA NRRL B-24137, further studies are needed on different application methods and field experiments are also required to establish an effective biocontrol agent.

## Conclusion

This study demonstrated the SA efficiency in decreasing the cotton wilt pathogen both *in vitro* and *in vivo*. Use of SA NRRL B-24137 might be considered as an ideal and effective biocontrol agent against soil-borne pathogens and to enhance the growth of cotton plants. One of the preferable alternatives to chemical control for sustainable agriculture is biological management of plant diseases. Therefore, more study should be focused on improving biocontrol strategies for soil-borne illnesses and determining the feasibility of such agents under harsh environmental circumstances.

##  Supplemental Information

10.7717/peerj.14754/supp-1 Supplemental Information 1 Inhibitory effect of SA NRRL B-24137 on spore germination of FOV

10.7717/peerj.14754/supp-2 Supplemental Information 2 Antagonistic effect of pre-sowing seed treatment with the suspension of SA NRRL B-24137 on *Fusarium* cotton wilt disease severity

10.7717/peerj.14754/supp-3 Supplemental Information 3 Reduction of *F. oxysporum* f.sp. *vasinfectum* (FOV) FOV induced wilt in cotton plants* reduction* using *Sa. algeriensis* NRRL B-24137 (SA).

10.7717/peerj.14754/supp-4 Supplemental Information 4 Disease severity of *Fusarium* wilt of cotton under greenhouse conditions Disease severity after different time interval.

10.7717/peerj.14754/supp-5 Supplemental Information 5 Assessment of *Sa. algeriensis* NRRL B-24137 and* F. oxysporum* f.sp. *vasinfectum* population density in the soil Assessment of SA and* FOV* density in the soil. after one week.

10.7717/peerj.14754/supp-6 Supplemental Information 6 Evaluation of *F. oxysporum* f.sp. *vasinfectum* papulation density Population density from 3 to 9 weeks.

10.7717/peerj.14754/supp-7 Supplemental Information 7 Spore germination raw data Spore germination after 1 week.

## Additional Information and Declarations

### Data Availability

The following information was supplied regarding data availability: Raw data is available in the Supplemental Files.
